# pH-responsive polymeric nanoparticles for peptide delivery: Synergistic STING pathway activation enhances tumor immunotherapy

**DOI:** 10.1016/j.ijpx.2025.100412

**Published:** 2025-10-06

**Authors:** Mengjie Rui, Haidan Tang, Lianglai Gao, Yujiao Hu, Wenyan Liang, Yinfeng Li, Chunlai Feng

**Affiliations:** a School of Pharmacy, Jiangsu University, 301 Xuefu Road, Zhenjiang 212013, Jiangsu Province, China; b NHC Key Laboratory of Diagnosis and Therapy of Gastrointestinal Tumor, Gansu Provincial Hospital, Lanzhou 730000, China

**Keywords:** Anti-PD-L1 therapeutic peptide, Polymeric nanocarriers, pH-responsive drug delivery, Tumor immunotherapy, STING pathway

## Abstract

Peptides hold great promise in tumor immunotherapy, but suffer from poor stability and short systemic circulation. To overcome these challenges, we developed a pH-responsive nanodelivery systems (P-NPs) based on the amphiphilic block polymer PEO-PC7A. In addition to its role in peptide encapsulation and protection, PEO-PC7A intrinsically acted as a stimulator of the interferon genes (STING) agonist, activating the cGAS-STING signaling pathway and remodeling the immunosuppressive tumor microenvironment. P-NPs were successfully prepared *via* a self-assembly technique, yielding nanoparticles with a uniform diameter of 91.2 ± 3.5 nm. Their pH-responsive behavior was confirmed by significant change in particle size and accelerated peptide release under acidic conditions. *In vitro*, P-NPs effectively increased the cytotoxic activity of T cells and induced higher interleukin-2 (IL-2) secretion compared to free peptide. In a 4 T1 tumor-bearing mouse model, intravenous administration of P-NPs achieved greater tumor growth inhibition and higher intratumoral interferon-γ (IFN-γ) levels than free peptide, with minimal systemic toxicity and no significant impact on body weight. Overall, our study presented a novel multifunctional peptide nanocarrier that enhanced tumor immunotherapy efficacy by concurrently improving peptide delivery and stimulating innate immunity, providing a promising foundation for the further development of innovative combination cancer immunotherapy strategies.

## Highlights

• A pH-responsive polymeric nanocarrier (PEO-PC7A) enables tumor-specific anti-PD-L1 peptide release under acidic conditions.
• The carrier polymer itself acts as a STING agonist, providing innate immune activation alongside checkpoint blockade.
• Dual-functional nanoplatform enhanced IL-2 and IFN-γ production, driving robust T cell activation and antitumor immunity.
• *In vivo* 4T1 orthotopic mouse model confirmed superior tumor suppression, immune remodeling with excellent systemic safety.
• Single-component nanoplatform offers a simplified yet effective strategy for combination immunotherapy.

## Graphical Abstract

**Figure:**
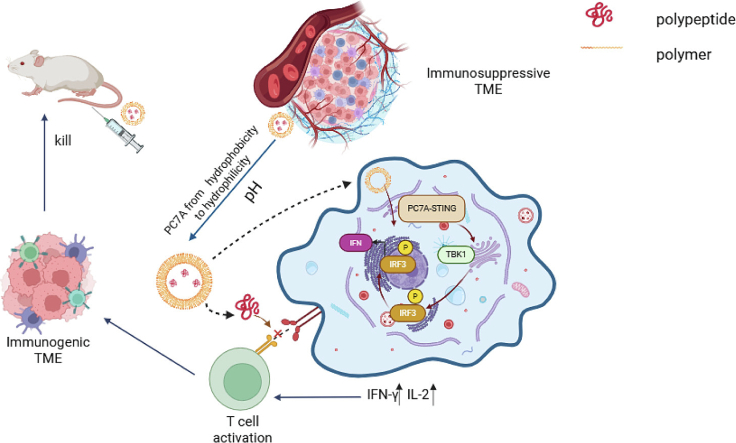

## 1 Introduction

Tumors represent one of the most life-threatening diseases(Bray et al., 2024; Guo et al., 2020; Hua et al., 2019; Yang et al., 2022). Traditional treatments include surgery, chemotherapy and radiotherapy. Surgical resection is only applicable to local tumors and has limited effect on metastatic or unresectable cancers; while chemotherapy and radiotherapy can kill cancer cells, but are accompanied by normal cell damage, serious side effects and drug resistance problems(Cashin et al., 2016; De Ruysscher et al., 2019; Li et al., 2021b).

Driven by advances in cancer immunology, immunotherapy has emerged as a transformative modality(Cheng et al., 2020). Cancer immunotherapy harnesses the host immune system to recognize and eliminate malignant cells, which offers the benefits of enhanced specificity, reduced side effects and sustained response compared to traditional modalities(Chen and Mellman, 2013; Chiang et al., 2025; Murciano-Goroff et al., 2020; Yalamandala et al., 2024). Among tumor immunotherapies, immune checkpoint blockade (ICB), particularly the inhibition of PD-1/PD-L1 pathway, has achieved remarkable success by reversing tumor immune suppression and recovering the effector function of exhausted T cells(Barclay et al., 2018; Huynh et al., 2025; Tan et al., 2020). Several monoclonal antibodies targeting PD-1 or PD-L1 have been approved for treating a range of cancer, including melanoma, non-small cell lung cancer, lung cancer, and others(Singh et al., 2020). Despite their success, the high production cost, inconvenient storage, and transportation of monoclonal antibodies limit their clinical application.

Given these limitations, peptide-based immune checkpoint inhibitors have gained increasing interest. These agents offer several advantages, including improved tissue penetration, favorable biocompatibility, and ease of synthesis(Liu et al., 2019; Varanko et al., 2020). Indeed, a number of PD-1/PD-L1 blocking peptides have been developed. Unfortunately, many such peptides exhibit low binding affinity and poor stability in the body, which result in rapid degradation and a short circulating half-life, limiting their therapeutic efficacy(Hossen et al., 2019). In particular, our previously developed peptide that blocked PD-1/PD-L1 interaction suffered from the enzymatic degradation in blood circulation, which certainly limited its anti-tumor efficacy(Rui et al., 2023). Therefore, designing a targeted peptide delivery system to improve the pharmacokinetic profile and tumor accumulation of our peptide is important for unlocking its full therapeutic potential.

Advances in nanotechnology and precision medicine have driven the development of nano drug delivery systems for cancer therapy(Gao et al., 2019; Maniam et al., 2018; Moorthy et al., 2025; Wang et al., 2025). Nanocarriers protect drugs from degradation, enhance solubility and prolong circulation time, thereby enhancing bioavailability and tumor-targeted accumulation while reducing off-target toxicity(Chen et al., 2014; Deng et al., 2012; Hu et al., 2012; Mura et al., 2013; Rösler et al., 2001). In particular, stimuli-responsive nanocarriers that respond to specific features of the tumor microenvironment have become a research hotspot in the past decade(Bapat et al., 2011; Li et al., 2012; Yalamandala et al., 2024; Yuan et al., 2020). One well-known feature of the tumor microenvironment is an acidic pH, typically ranging from pH 6.2 to 6.8 due to the high glycolytic metabolism of tumor cells, compared to normal blood and tissue pH of 7.2–7.4(Helmlinger et al., 2002; Tang et al., 2011). To exploit this difference, many pH-responsive polymers have been designed for cancer drug delivery, remaining inert at physiological pH but rapidly releasing their drug payload under acidic conditions to achieve targeted anti-tumor effects(Gillies and Fréchet, 2003; Stubbs et al., 2000). Generally, pH-responsive polymeric carriers are engineered *via* two main strategies: (1) incorporating acid-cleavable linkages or pH-sensitive non-covalent interactions to attach the drug, (2) using polymer structures that undergo protonation or bond cleavage in acidic pH to trigger carrier disassembly and payload release(Che et al., 2016; Deirram et al., 2019; Lv et al., 2018). Functional groups such as acetal, orthoester, hydrazone, imine, vinyl ether, succinimide have been widely explored for this purpose(Ali et al., 2006; Aryal et al., 2010; Gillies et al., 2004; Griset et al., 2009; Wang et al., 2008).

**Scheme 1:**
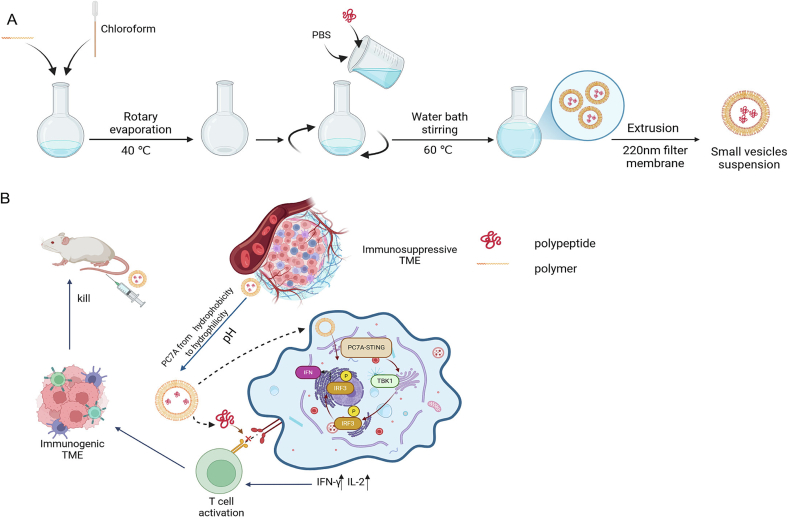
Schematic illustration of the composition of peptide-loaded pH-responsive nanoparticles (P-NPs), as well as the processes of charge reversal and shape transformation, and the mechanism of synergistic immunotherapy.

In this study, we designed a straightforward but robust delivery nanoparticle to improve the therapeutic efficacy of our anti-PD-L1 peptide. A central component of this system is a pH-responsive amphiphilic block copolymer, PEO-PC7A, which self-assembles into polymeric vesicles in aqueous solution. As illustrated in Scheme 1A, the hydrophilic anti-PD-L1 peptide (Rui et al., 2023) was encapsulated within the aqueous core of these particles, generating a targeted peptide delivery system. Notably, PC7A-based polymers have been reported as immunoadjuvants that potentiate antitumor immunity(Li et al., 2021a; Luo et al., 2017); however, these implementations combine adjuvanticity with separate cargoes or rely on vaccination-style antigens. Here, we engineered a single-material nanoplatform that unified two coordinated functions (Scheme 1B): (i) acid-triggered delivery of the anti-PD-L1 peptide and (ii) intrinsic activation of the cyclic GMP-AMP synthase (cGAS)-STING pathway by the PC7A domain. By explicitly mapping polymer protonation to nanostructure transitions and pH-dependent release, we spatiotemporally gate immune activation to acidic tumor and endo/lysosomal milieus. Collectively, this dual-function strategy aims to achieve synergistic therapeutic benefits by simultaneously enhancing checkpoint blockade efficacy and stimulating immunomodulatory signaling.

## 2 Materials and Methods

### 2.1 Materials

2-(Azepan-1-yl)ethanol was obtained from Shanghai Bide Pharmaceutical Technology Co. Ltd. (Shanghai, China). Methacryloyl chloride was obtained from Adamus Reagent Co. Ltd. (Shanghai, China). Petroleum ether, ethyl acetate, 2-bromoisobutyryl bromide, triethylamine (TEA), 4-dimethylaminopyridine (DMAP), N,N,N′,N″,N″-pentamethyldiethylenetriamine (PMDETA), and anhydrous tetrahydrofuran (THF), were purchased from Aladdin Reagents Co. Ltd. (Shanghai, China). Poly(ethylene glycol) monomethyl ether (mPEG, Mw 5000) was purchased from Shanghai McLean Biochemistry Technology Co. Ltd. (Shanghai, China). All other solvents and chemical reagents were of analytical grade and used as received unless otherwise noted.

The anti-mouse monoclonal antibodies CD8 (Cat #ab217344), GZMB (Cat # ab4059), and FOXP3 (Cat #ab253297) were purchased from Abcam (San Diego, CA, USA). Rabbit polyclonal pTBK1 (Ser172) (Cat #AF8190) was obtained from Affinity Biosciences. Mouse anti-IgG, HRP-linked antibody was procured from Abcam (San Diego, CA, USA).

The anti-PD-L1 peptide (sequence: DWFKAFYDKINETYNK) was synthesized following standard Fmoc solid-phase peptide synthesis protocols by GL Biochem Ltd. (Shanghai, China) and provided at a purity of greater than 95 %(Rui et al., 2023).

The human T-lymphoblastoid cell line (Jurkat), human breast cancer cell line (MDA-MB-231), murine breast cancer cell line (4 T1), and human non-tumorigenic epithelial cell line (MCF10A) were obtained from the Type Culture Collection of the Chinese Academy of Sciences (Shanghai, China).

The Jurkat and 4 T1 cells were maintained in Roswell Park Memorial Institute 1640 medium (Cytiva HyClone, UT, USA) supplemented with 10 % fetal bovine serum (FBS; Sijiqing, Hangzhou, Zhejiang, China), 1 % penicillin-streptomycin (Beyotime, Shanghai, China), and 1 % l-glutamine. MDA-MB-231 cells were cultivated in Dulbecco's Modified Eagle Medium (DMEM, Cytiva HyClone) with 10 % FBS and 1 % penicillin-streptomycin. MCF10A cells were cultivated in DMEM/F12 (1:1, Cytiva HyClone) with 10 % FBS, 1 % penicillin-streptomycin, 100 μg/mL epidermal growth factor (EGF), 1 mg/mL hydrocortisone, and 10 mg/mL insulin. All cell cultures were maintained in a humidified incubator at 37 °C with 5 % CO_2_.

Female BALB/c mice (5–6 weeks old, 20 g) were purchased from the Laboratory Animal Center of Jiangsu University. All animals were housed in a pathogen-free facility with free access to food and water. Animal studies were conducted in accordance with institutional guidelines and approved by Institutional Animal Care and Use Committee of Jiangsu University.

### 2.2 Synthesis and Characterization of Amphiphilic Block Polymer PEO-PC7A

**Fig. 1:**
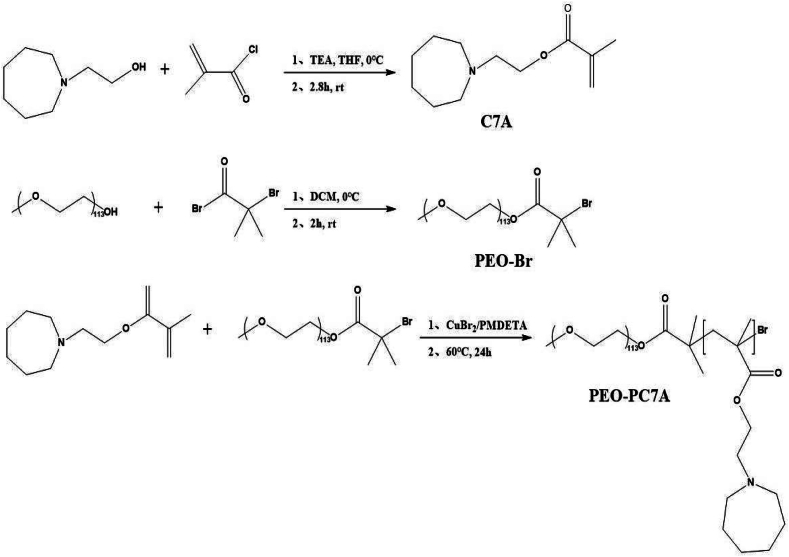
Synthesis route of the amphiphilic block polymer PEO-PC7A.

The amphiphilic block polymer PEO-PC7A was synthesized as illustrated in Fig. 1. The synthesized products were confirmed by ^1^H NMR on a 400 MHz NMR spectrometer (Bruker GmbH, Germany) and Fourier infrared spectrometer (FTIR, AVATAR-370, Nicolet Instrument).

#### 2.2.1 Synthesis of monomer C7A

2-(Azepan-1-yl)ethanol (2.0 g, 14 mmol) was dissolved in anhydrous THF (30 mL) in a round-bottom flask, followed by the addition of TEA (28 mmol, 4 mL). The solution was cooled to 0 °C in an ice bath. A solution of methacryloyl chloride (2.4 mL, 25.2 mmol) in anhydrous THF (10 mL) was added dropwise *via* a constant-pressure dropping funnel under stirring. The mixture was stirred at room temperature for 10 h. The precipitated triethylamine hydrochloride was removed by filtration, and the solvent was evaporated under reduced pressure. The crude product was purified by column chromatography on silica gel (petroleum ether/ethyl acetate = 4/1, *v*/v) to obtain a light-yellow oil identified as 2-(hexamethylenimino)ethyl methacrylate (C7A).

#### 2.2.2 Synthesis of macromolecular initiator PEO-Br

Methoxy poly(ethylene glycol) (mPEG, 3.0 g, 0.6 mmol) was dissolved in anhydrous dichloromethane (DCM, 20 mL). TEA (0.42 mL, 3 mmol) and DMAP (73 mg, 0.6 mmol) were added dropwise. The mixture was cooled to 0 °C. 2-bromoisobutyryl bromide (0.3 mL, 2.4 mmol) dissolved in anhydrous THF (10 mL) was added dropwise at 0 °C under stirring. The stirring continued at room temperature for 24 h. The solution was terminated and then washed with saturated sodium bicarbonate solution. The aqueous phase was extracted three times with DCM (3 × 50 mL). The combined organic layers were dried over anhydrous Na_2_SO_4_, filtered, and concentrated. The concentrate was precipitated into cold diethyl ether and dried under vacuum at 35 °C to yield a white solid, the bromine-terminated macromolecular initiator PEO—Br.

#### 2.2.3 Polymerization of amphiphilic PEO-PC7A block copolymer

PEO-Br (200 mg, 0.04 mmol) and monomer C7A (700 mg, 3.2 mmol) were introduced into a three-necked flask, followed by the addition of isopropanol (1 mL) and *N*,*N*-dimethylformamide (DMF, 1 mL). PMDETA (104 mg, 0.6 mmol) was then added as a ligand, and copper(I) bromide (CuBr, 4.5 mg, 0.02 mmol) as the catalyst for atom transfer radical polymerization (ATRP). The system was deoxygenated *via* nitrogen for 20 min (alternating vacuum and nitrogen three times to remove oxygen). Subsequently, deoxygenated ascorbic acid (105 mg, 0.6 mmol) was added to initiate polymerization, and the reaction was carried out at 60 °C for 24 h. After that, the polymerization was terminated by exposing the reaction to air. The solution was diluted with THF (30 mL) and passed through a neutral alumina column to remove copper catalyst. The solvent was evaporated under reduced pressure, and the resulting product was dialyzed against ultrapure water (MWCO 3.5 kDa) for 24 h to eliminate residual monomers. Finally, PEO-PC7A copolymer was obtained by freeze-dried.

#### 2.2.4 Determination of critical Micelle Concentration (CMC)

The CMC value of the synthesized PEO-PC7A copolymer was investigated using the pyrene fluorescent probe method. A stock pyrene solution (0.4 mM) was prepared in acetone and stored in the dark. A gradient of aqueous PEO-PC7A solutions with varying concentrations (5 × 10^−6^, 1 × 10^−5^, 2.5 × 10^−5^, 5 × 10^−5^, 1 × 10^−4^, 1 × 10^−3^, 1 × 10^−2^, 2.5 × 10^−2^, 5 × 10^−2^, 0.1, 0.2 mg/mL) were prepared. To each solution, the pyrene acetone solution (10 μL) was added to achieve a final concentration of pyrene of 1 μM.

The mixtures were incubated at room temperature for 2 h, and then left in the dark overnight to equilibrate. Fluorescence excitation spectrum of each sample was recorded from 300 nm to 370 nm using a fluorescence spectrophotometer (with emission monitored at 390 nm). Excitation and emission slit widths of 3 nm and 5 nm, respectively. The CMC value was identified from the plot of the intensity ratio *versus* the logarithmic concentration of PEO-PC7A, as the concentration at with a sharp increase in the ratio occurred, corresponding to the nanoparticle formation.

#### 2.2.5 pH titration

The PEO-PC7A copolymer was first dissolved in HCl solution (0.1 M) to prepare a stock solution at a concentration of 1 mg/mL. pH titration was carried out by adding 20 μL increments of 0.1 M NaOH solution under stirring. The pH values were measured by a pH meter. The increase in pH in the range of 3–11 was monitored as a function of the total added volume of NaOH (V_NaOH_).

### 2.3 Preparation and characterization of peptide-loaded nanoparticles (P-NPs)

#### 2.3.1 Preparation of P-NPs

P-NPs were prepared using a thin-film hydration method. Briefly, PEO-PC7A copolymer (10 mg) was dissolved in chloroform (1 mL). The organic solvent was evaporated by a rotary evaporator at 40 °C, forming a uniform then film of copolymer on the flask wall. The film was subsequently hydrated with a phosphate-buffered saline solution (PBS, 10 mM, pH 7.4) containing the anti-PD-L1 peptide at 60 °C for 5 h with occasional gentle agitation. Finally, the resulting nanoparticle suspension was extruded 11 times through 200 nm polycarbonate membrane using an Avanti mini extruder. After extrusion, the samples were transferred into a dialysis bag (MWCO: 3 kDa) and immersed in 250 mL of PBS, stirring for 2 h to remove free peptide. The final P-NPs suspension was stored at 4 °C until use.

#### 2.3.2 Characterization

The effective particle diameters, polydispersity index (PDI), and zeta potentials of the P-NPs samples were measured using a laser particle size analyzer (Malvern Zetasizer Nano ZS, UK) at 25 °C. The morphology of the P-NPs was observed by JEOL JEM-1230 transmission electron microscopy (TEM) (Japan). For TEM analysis, a droplet of the P-NP suspension was deposited onto a carbon-coated copper TEM grid, allowed to adsorb for 1 min, and excess liquid was blotted off. The sample was then negatively stained with 1 % phosphotungstic acid, dried, and imaged under the TEM.

#### 2.3.3 Effect of pH on particle size

To assess the pH-responsive behavior of the P-NPs, changes in particle size under neutral and acidic conditions were evaluated. Aliquots of the P-NP solution (1 mL) were mixed with 10 mL of appropriate buffer at pH 7.4 (phosphate buffer) or pH 6.5 (phosphate buffer) or pH 5.5 (citri acid-phosphate buffer) and incubated at room temperature for 48 h. At appropriate time points, samples were withdrawn and analyzed by DLS to determine their size distribution, PDI, and zeta potential. The changes in particle size, PDI, and zeta potential served as indicators of nanoparticle stability or structural disassembly under different pH conditions.

#### 2.3.4 *In vitro* peptide release

The *in vitro* release profile of the encapsulated peptide from P-NPs was evaluated at pH 7.4, pH 6.5, and pH 5.5 using a dialysis method. P-NPs suspensions (1 mL) were added to in dialysis bags (MWCO: 10 kDa) and placed in various PBS release medium (100 mL) at the corresponding pH. The dialysis was conducted under stirring at 37 °C (100 rpm). At appropriate time points (0.5, 1, 2, 4, 8, 12, 24, 48, 72 h), 0.5 mL of external release medium was withdrawn and immediately replaced with an equal volume of fresh PBS of the same pH. Peptide concentrations in the collected aliquots were quantified by HPLC method, and cumulative release was expressed as a percentage of the total peptide encapsulated.

### 2.4 *In vitro* studies

#### 2.4.1 PHA activation of Jurkat cells

To establish the optimal concentration of phytohemagglutinin (PHA) for activating Jurkat T cells, we evaluated Jurkat cell proliferation and IL-2 secretion in response to various PHA concentrations. Jurkat cells were seeded in 96-well plates at 4 × 10^4^ cells/well in 100 μL and cultured for 24 h. The cells were then treated with medium containing PHA at final concentrations of 0.5, 1, 2, 4, or 8 μg/mL. After 48 h of incubation with PHA, cell viability was determined using the Cell Counting Kit-8 (CCK-8) assay. CCK-8 reagent (20 μL) was added to each well and incubated with the cells for 1.5 h, after which the absorbance at 450 nm (OD_450_) was measured using a microplate reader. In parallel, culture supernatants were collected from each well by centrifuging the plates at 300 ×g for 10 min to pellet cells and analyzed for IL-2 levels using an IL-2 ELISA kit (MultiScience, Hangzhou, China). The IL-2 concentration in the supernatants served as an indicator of T-cell activation. The PHA concentration that achieved high IL-2 secretion with minimal cytotoxicity was identified and used in subsequent co-culture experiments.

#### 2.4.2 Cell viability assay

The cell viabilities were conducted to evaluate two aspects of cytotoxicity *in vitro*: (a) the cytocompatibility of blank (peptide-free) PEO-PC7A vesicles on cancer cells, and (b) the anti-tumor effect of peptide-loaded P-NPs in a co-culture model of tumor cells and Jurkat cells.

To assess the cytotoxicity of PEO-PC7A nanoparticles, three cell lines, including human MDA-MB-231 breast cancer cell, murine 4 T1 breast cancer cell, and human MCF10A non-tumorigenic epithelial cell were seeded separately into 96-well plates at a density of 5 × 10^3^ cells/well and then cultured for 24 h. The medium was then replaced with fresh medium containing blank PEO-PC7A nanoparticles at 25, 50, 100, 200, or 400 μg/mL (polymer concentration). After 48 h of treatment, cell viability was investigated by the CCK-8 assay.

To mimic the interaction between tumor cells and T lymphocytes, a co-culture system was established using MDA-MB-231 breast cancer cells and Jurkat cells. Prior to co-culture, MDA-MB-231 cells were incubated with human interferon-γ (IFN-γ, 10 ng/mL) for 24 h to upregulate PD-L1 expression, whereas Jurkat cells were activated with PHA (1 μg/mL) for 48 h. To assess the baseline IL-2 level, unstimulated Jurkat cells were used as control. The pre-treated MDA-MB-231 cells were seeded into 96-well plates and allowed to attach for 24 h, after which the medium was replaced with fresh DMEM. PHA-activated Jurkat cells or unstimulated Jurkat cells were subsequently added (4 × 10^4^ cells per well). Control wells containing MDA-MB-231 cells alone (without Jurkat cells) were included to assess baseline tumor cell viability. Various concentrations of free peptide and peptide-loaded P-NPs (corresponding to 12.5, 25, 50, 100, 150 μg/mL of peptide dose) were added to the co-culture wells. Following 48 h of co-incubation, wells were washed gently with PBS to remove residual drug and suspended Jurkat cells. Adherent cells were then incubated with CCK-8 working solution (10 % in medium, 100 μL) for 2 h at 37 °C, and absorbance at 450 nm was recorded to determine the viability of MDA-MB-231 tumor cells.

To further investigate how P-NPs activate T cells, various concentrations of PEO-PC7A polymer, free peptide and peptide-loaded P-NPs (corresponding to 12.5, 25, 50, 100, 150 μg/mL of peptide dose) were added to the co-culture wells. After incubation, IL-2 levels in the co-culture supernatants were measured using the IL-2 ELISA kit.

### 2.5 *In vivo* animal studies

#### 2.5.1 *In vivo* tissue distribution of P-NPs

To assess the biodistribution of the therapeutic peptide and its nanoparticle formulation, an *in vivo* imaging experiment was conducted using Sulfo-Cyanine7 (Cy7)-labeled peptide. Cy7-labeled peptide (Cy7-P) and Cy7-labeled peptide-loaded nanoparticles (Cy7-P-NPs) were prepared for fluorescence tracking.

To establish a breast cancer tumor model, 4 T1 cells (1 × 10^7^) were injected subcutaneously into female BALB/c mice (5–6 weeks old, 20 g). When tumors reached an approximate volume of 100 mm^3^, mice were intravenously injected with 0.2 mL of either Cy7-P or Cy7-P-NPs, with equivalent amount of peptide. At 12 h and 24 h post-injection, the mice were anesthetized and imaged using an *In Vivo* fluorescence imaging system to track the distribution of the fluorescent signal. Following the final imaging time, mice were euthanized and tumors along with major organs, including heart, liver, spleen, lung, and kidney, were harvested. These tissues were immediately imaged *ex vivo*, and fluorescence intensities were quantified to compare tissue distribution profiles of free peptide *versus* nanoparticle-encapsulated peptide.

#### 2.5.2 *In vivo* antitumor efficacy

The antitumor efficacy of P-NPs was investigated in an orthotopic 4 T1 breast tumor model. Female BALB/c mice were inoculated in the mammary fat pad with 0.15 mL of 4 T1 cell suspension (1 × 10^7^ cells/mL). When individual tumor volume reached 50–100 mm^3^, the mice were randomly divided into five groups (*n* = 5 per group): (1) saline control, (2) blank polymer vesicles (no peptide, equivalent polymer dose of 20 mg/kg), (3) free peptide (20 mg/kg), (4) low-dose P-NPs (10 mg/kg peptide), and (5) high-dose P-NPs (20 mg/kg peptide).

To augment immune activation within the tumor, all mice were pre-treated with intratumoral injections of murine IFN-γ (0.2 mL of 0.75 μg/mL), administered once daily for three consecutive days, starting on day 5 after tumor inoculation. The therapeutic treatments were then given intravenously through the tail vein every other day for a total of six injections once this priming had been completed. During the course of treatment, measurements of tumor volumes and body weight were taken at regular intervals. Tumor size was measured with calipers in two perpendicular dimensions, and the corresponding volume was calculated by employing the formula (width^2^ × length)/2.

At the end of the treatment schedule, all mice were euthanized. Tumors were excised, weighed, and compared among groups. Major organs were collected for macroscopic examination. To evaluate the immune response in the tumor microenvironment, tumor tissues were homogenized and the level of IFN-γ in tumor lysates from each group was measured using an ELISA kit. Increased intratumoral IFN-γ was used as an indicator of effective T cell activation and an immunostimulatory tumor microenvironment.

After euthanasia the tumors were collected, fixed, paraffin-embedded, and sectioned. Afterwards, the expression of the indicated proteins was detected by a horseradish peroxidase detection system, and sections were examined by microscopy.

### 2.6 Statistical analysis

All data are presented as the mean ± standard deviation (SD). Two groups were compared using an unpaired, two-tailed Student's *t*-test, and multiple groups were compared using one-way analysis of variance (ANOVA) followed by Tukey's *post hoc* test. A *p*-value of less than 0.05 was considered significant. All analyses were performed using GraphPad Prism (version 8.0).

## 3 Results and discussion

### 3.1 Characterization of Amphiphilic Block Polymer PEO-PC7A

#### 3.1.1 ^1^H NMR spectroscopy

**Fig. 2:**
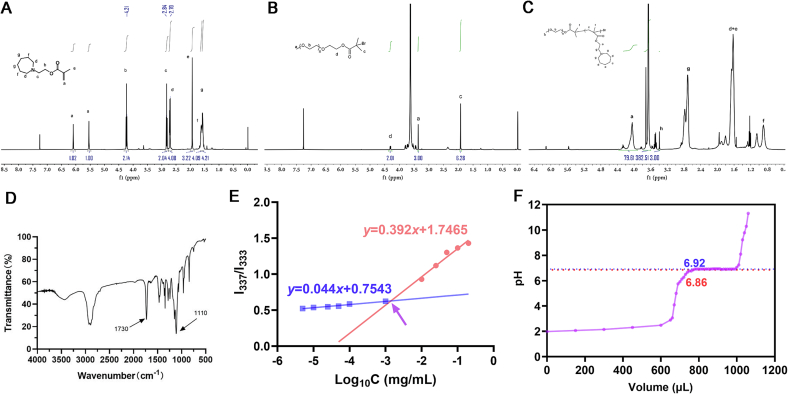
Structural characterization of the polymer and CMC determination of PEO-PC7A. (A-C) ^1^H NMR spectra of (A) monomer C7A, (B) macroinitiator PEO—Br, and (C) block copolymer PEO-PC7A in CDCl_3_. (D) FT-IR spectrum of PEO-PC7A, showing characteristic absorption bands (highlighted) for C—H, C=O, and C—N bonds. (E) Pyrene fluorescence method for CMC determination. The plot of I_337_/I₃₃₃ *versus* log_10_[PEO-PC7A] demonstrated two linear regimes (unassembled *vs* post-assembly). (F) pH titration of PEO-PC7A; the inflection around pH 6.9 indicated the apparent p*K*_a_ of the PC7A segment.

The structures of the synthesized monomer C7A, brominated initiator PEO—Br, and the block copolymer PEO-PC7A were confirmed by ^1^H NMR spectroscopy. The ^1^H NMR spectrum of the C7A monomer in CDCl_3_ was shown in Fig. 2A. As a result, vinyl protons of C7A appear as peak a at *δ* 5.5 and 6.0 ppm, and the methyl proton of the methacrylate appears as peak e at *δ* 1.9 ppm. Peaks d, f, and g at *δ* 2.71, 1.6, and 1.57 ppm, respectively, correspond to methylene protons on the ring of C7A. Peaks b (*δ* 4.2 ppm) and c (*δ* 2.8 ppm) were shifted downfield due to the electron-withdrawing effects of the adjacent oxygen and nitrogen atoms in the monomer's structure. The integral ratio of peak a to peak b was approximately 1:1, consistent with the structure of C7A, confirming its successful synthesis.

The ^1^H NMR spectrum of the brominated initiator PEO-Br was shown in Fig. 2B. The peaks a and b at *δ* 3.4 and 3.7 ppm corresponded to the methylene and methoxy protons of the poly(ethylene oxide) chain. Peaks c and d at *δ* 1.9 and 4.3 ppm were new signals characteristic of the α-bromoester end group introduced into PEO—Br. Specifically, peak c represented the two methyl protons of the bromoester, and peak d was the methylene proton adjacent to the ester oxygen. The appearance of these new peaks, and their integration ratio relative to the PEO main-chain peaks (ratio of c:d:a ≈ 6:3:2), indicated successful functionalization of mPEG to form the macroinitiator PEO—Br.

After polymerization of C7A from the PEO-Br initiator, the ^1^H NMR spectrum of the resulting block copolymer PEO-PC7A contained signals from both components (Fig. 2C). Peaks d, e, and g (at *δ* 1.63 and 2.71 ppm) corresponded to the methylene protons on the nitrogen-containing seven-membered ring in the PC7A block. Notably, the protons at position g were slightly further downfield due to the electron-withdrawing effect of the adjacent protonated nitrogen atom. Peaks b and f (*δ* 3.7 and 1.9 ppm) were still observed from the PEO-Br segment (methylene and terminal methyl protons, respectively). The presence of all these signals confirmed that the PEO and PC7A blocks have been successfully connected to form PEO-PC7A. Based on the integration of representative peaks, the degree of polymerization of the PC7A block was calculated to be approximately 40 repeating units.

#### 3.1.2 Fourier transform infrared spectroscopy (FT-IR)

The chemical structure of PEO-PC7A was further verified by FT-IR (Fig. 2D). The spectrum showed strong C—H stretching bands at 2800–3000 cm^−1^, attributable to methyl and methylene groups in the aliphatic backbone. A prominent absorption at ∼1730 cm^−1^ corresponded to the ester C=O stretch, confirming the presence of methacrylate-derived ester linkages. Additional bands included 1460–1470 cm^−1^ (CH_2_ scissoring), 1340–1380 cm^−1^ (CH_3_ bending), and a shoulder near 1245–1265 cm^−1^ assigned to the ester C—O stretch. A strong band at ∼1110 cm^−1^ arose from overlapping PEO ether (C-O-C) stretching and tertiary amine C—N stretching from the seven-membered ring in the PC7A segment, consistent with incorporation of the amine-bearing block. A broad, low-intensity feature near ∼3400 cm^−1^ is attributed to O—H stretching from residual moisture and/or terminal hydroxyl groups. The overall pattern, in agreement with the ^1^H NMR data, confirmed the expected functional groups of the PEO-PC7A copolymer.

#### 3.1.3 Determination of CMC

The CMC of PEO-PC7A was determined using pyrene fluorescence method (Fig. 2E). As pyrene transferred from aqueous to a hydrophobic polymeric microenvironment, the emission band intensities at 337 nm and 333 nm changed; therefore, the ratio I_337_/I_333_ sensitively reported the assembly of PEO-PC7A. Plotting I_337_/I_333_ ratio against log_10_[polymer] produced two linear regimes corresponding to unassembled chains at low concentrations and assembled nanostructures at higher concentrations. The intersection of these fits yielded a CMC of 1.41 × 10^−3^ mg/mL, demonstrating a strong propensity to self-assemble at dilute conditions. This low CMC supported the dilution stability of P-NPs and revealed robust assemblies in physiological buffer.

#### 3.1.4 pH titration

To further investigate the pH responsiveness of PeO-PC7A, we performed pH titration. As shown in Fig. 2F, upon continuous dropwise addition of NaOH solution, the titration curve displayed a sharp turn at approximately pH 6.8, followed by a plateau. This phenomenon reflects the deprotonation of the tertiary ammonium groups on the PC7A segment and is in accordance with the apparent p*K*_a_ value of PC7A(Zhou et al., 2011), approximately 6.8. This transition indicated that small pH decreases into the mildly acidic range are sufficient to alter the ionization state of PC7A and increase its hydrophilicity, thereby destabilizing the nanoparticles in the tumor microenvironment.

### 3.2 Preparation and characterization of P-NPs

#### 3.2.1 Characterization of P-NPs under physiological condition

**Fig. 3:**
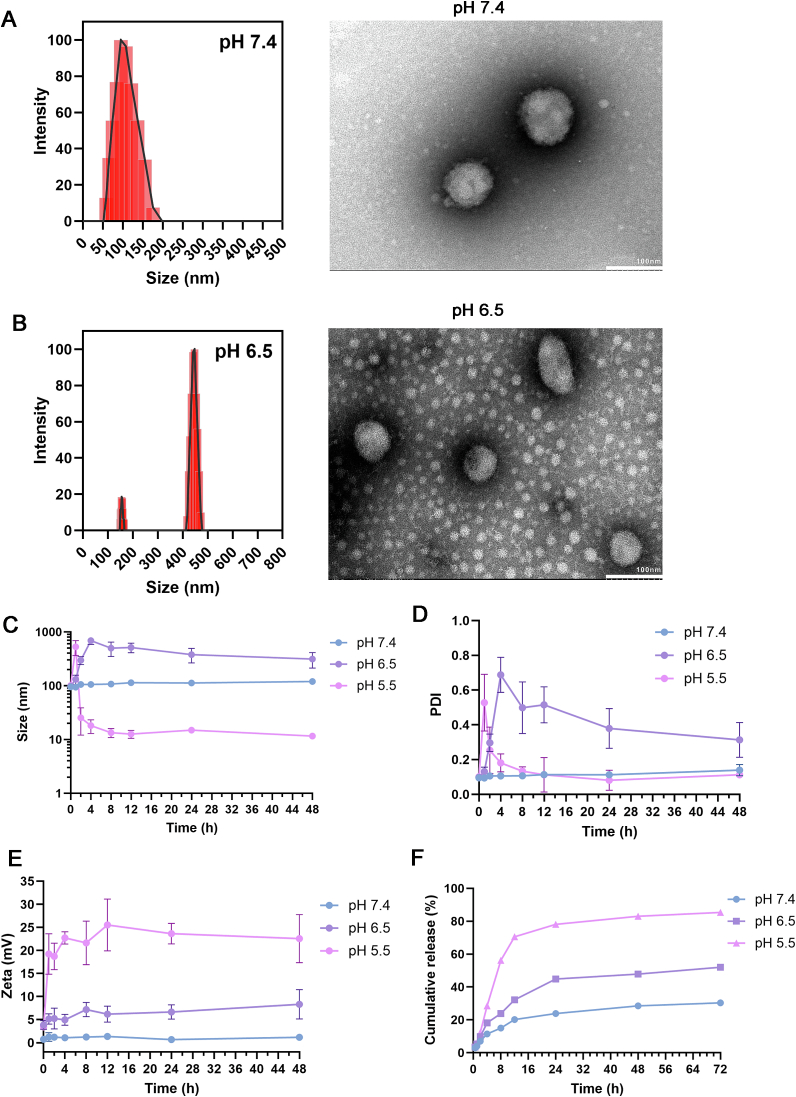
Physicochemical characterization and pH-responsive behavior of peptide-loaded P-NPs. (A) Dynamic light scattering (DLS) size distribution and TEM image of P-NPs under physiological condition (pH 7.4), showing a monodisperse population with an average diameter around 90 nm and spherical morphology. (B) DLS and TEM characterization of P-NPs in mildly acidic environment (pH 6.5), showing enlarged particles together with a population of smaller fragments. (C-E) Time-dependent changes in (C) particle size, (D) polydispersity index, and (E) zeta potential of P-NPs at pH 7.4, 6.5, and 5.5 over 48 h. (F) Cumulative peptide release profiles from P-NPs over time at pH 7.4, 6.5, and 5.5 (37 °C).

Peptide-loaded nanoparticles (P-NPs) were prepared by thin-film hydration. As shown in Fig. 3A, P-NPs under physiological condition (pH 7.4) exhibited an average hydrodynamic diameter of 91.2 ± 3.5 nm with a PDI of 0.187 ± 0.016, indicating a relatively narrow size distribution. The sub-100 nm diameter is favorable for tumor accumulation *via* the enhanced permeability and retention (EPR) effect. The DLS particle size distribution was normalized such that the maximum peak intensity corresponds to 100 %, facilitating visual comparison of particle populations across sizes. The area under the curve did not represent absolute percentage but reflected the relative distribution and width of particle sizes.

TEM imaging revealed well-defined spherical or slightly spheroidal polymeric nanoparticles with no evidence of aggregation (Fig. 3A, right). Notably, particle diameters observed by TEM were slightly smaller than the DLS-measured values, which is expected due to dehydration and collapse of soft nanostructures during TEM sample preparation. Furthermore, when incubated at pH 6.5, P-NPs demonstrated a significant increase in apparent size relative to pH 7.4, along with a broader and multimodal distribution (Fig. 3B). TEM images revealed both enlarged particlesand a population of smaller fragments, consistent with partial disassembly/restructuring of the assemblies. Mechanistically, these observations were attributed to the ionization transition of PC7A near its p*K*_a_ and the protonation of the tertiary amines increased polymer hydrophilicity and electrostatic repulsion, disrupting the amphiphilic balance required for nanoparticle stability. While the spherical morphology and pH responsiveness suggested a core-shell or vesicle-like structure, TEM imaging cannot unambiguously resolve internal aqueous compartments. Further structural elucidation by cryo-TEM or small-angle scattering is planned to confirm vesicle architecture.

#### 3.2.2 Effect of pH on particle size and surface charge

One key design feature of PEO-PC7A nanoparticles was their pH-responsive behavior. To evaluate their pH-responsive behavior, P-NPs were incubated in buffer at pH 7.4, 6.5, and 5.5 over 48 h, followed by particle size and zeta potential analysis (Fig.3C-E). At pH 7.4, particles remained approximately 100 nm with low PDI across the entire time course, confirming excellent colloidal stability in neutral media. At pH 6.5, the size of P-NPs increased progressively from initial 100 nm to 497 nm by 48 h, with a broadened, multimodal distribution (Fig. 3C-D). This result is in accordance with the TEM image at pH 6.5, implying coexistence of swollen assemblies and disassembled species. The zeta potential showed a modest positive shift, consistent with partial protonation of tertiary amines. When the pH decreased to 5.5, the apparent size decreased to approximately 10 nm range and the distribution narrowed (Fig. 3C-D), while the zeta potential increased to 22.6 ± 5.2 mV (Fig. 3E).

This pH-triggered behavior is attributed to the protonation of the tertiary amine groups of the PC7A segment under acidic conditions. Protonation increased the hydrophilicity of the polymer, disrupting the amphiphilic balance necessary for maintaining vesicle integrity. Consequently, the polymer's hydrophobic interactions within the core were weakened, leading to vesicle expansion, fusion, or breakdown. This transition from a stable nanostructure at physiological pH to an unstable state under acidic conditions supports the nanoparticles' ability to remain intact in the bloodstream while enabling targeted payload release in acidic environments such as the tumor microenvironment or intracellular endo/lysosomal compartments. These findings affirm the utility of PEO-PC7A as a responsive nanocarrier for precision drug delivery(Gao et al., 2023).

#### 3.2.3 *In Vitro* release of P-NPs

We next evaluated the release profile of encapsulated peptide under various pH conditions to further assess the pH-responsive behavior of P-NPs. Three pH, including pH 7.4, pH 6.5, and pH 5.5, were selected to mimic the relevant environments that P-NPs would encounter biologically. Specifically, pH 7.4 represented physiological blood or extracellular pH, pH 6.5 approximated the slightly acidic tumor microenvironment, and pH 5.0–5.5 corresponded to the late endosomal/lysosomal pH inside cells.

As expected, P-NPs displayed a pH-dependent release pattern (Fig. 3F). At physiological condition (pH 7.4), the cumulative release of the peptide over 72 h was limited to 30.4 %, indicating that the peptide remained substantially entrapped within the nanoparticles during circulation, which was a feature in reducing premature release and off-target exposure. When the pH was lowered to 6.5 and simulated the mildly acidic tumor microenvironment, the release rate increased significantly, with approximately 52.1 % of the peptide released over the same time period. Under the representative conditions of endosome and lysosome (pH 5.5), the peptide release was accelerated and near complete, reaching a cumulative release of 85.4 % in 72 h. Obviously, this enhanced release at lower pH was primarily attributed to the protonation of the tertiary amine groups in the PC7A block that have been discussed above.

Collectively, these results confirmed a protonation-driven structural transition in PEO-PC7A assemblies and its effect on peptide release. Under physiological pH, the PC7A segment is largely unprotonated and hydrophobic, sustaining tight packing of the polymer chains and efficient encapsulation of hydrophilic peptides within the vesicular core(Wang et al., 2020; Wang et al., 2014). In slightly acidic tumor microenvironment (pH 6.5), partial protonation increased the hydrophilicity of PC7A and introduced electrostatic repulsion within the hydrophobic domain. The amphiphilic balance is perturbed but not fully lost, triggering time-dependent swelling and permeability enhancement, thereby allowing a controlled peptide release. Upon further acidification to pH 5.5, substantial protonation of PC7A segment resulted in complete loss of the hydrophobic driving force, converting the polymer into a highly positive charged, water-soluble form. This transition leads to nanoparticle disassembly into polymer chains or small micellar fragments, as reflected by the decrease in particle size and increased zeta potential. As shown in Fig. 3F, this correlated with an initial burst release followed by sustained release over 72 h. This release kinetic profile reflected rapid diffusion from destabilized structures, followed by slower release from residual assemblies or dissolved polymer. Furthermore, the measurements were not extended below pH 5.5 because such conditions are uncommon during nanoparticle trafficking and pose higher risks of peptide degradation and buffer-related artifacts. Given the sharp protonation behavior of PC7A, further acidification would be expected to yield similar or faster release without additional mechanistic insight.

Overall, this pH-triggered mechanism could enhance therapeutic selectivity in acidic environments while maintaining systemic stability, thereby improving both efficacy and safety of the P-NPs.

### 3.3 *In vitro* studies

#### 3.3.1 PHA-mediated activation of Jurkat T cells

Jurkat cells, which is an established human T lymphocyte cell line, has been extensively utilized as a model system for investigating signaling pathways involved in T cell activation(Chen and Nong, 2018). The ability to selectively activate or inhibit these pathways in Jurkat cells allows for detailed investigation into the mechanisms that control T cell activation, differentiation, and cytokine production. For the purposes of our co-culture experiments, creating an immune-stimulated environment was a prerequisite. In their quiescent state *in vitro*, Jurkat cells exhibit negligible production of T-cell-associated cytokines. To induce an activated state, we employed phytohemagglutinin (PHA) to stimulate Jurkat cell(Li et al., 2020). The activation of these cells is commonly associated with the secretion of interleukin-2 (IL-2), a pivotal cytokine that promotes T-cell growth and orchestrates various functional responses. In this study, Jurkat cells were treated with a concentration gradient of PHA (0 to 8 μg/mL) for 48 h, after which IL-2 levels in the supernatant and cell viability were measured.

**Fig. 4:**
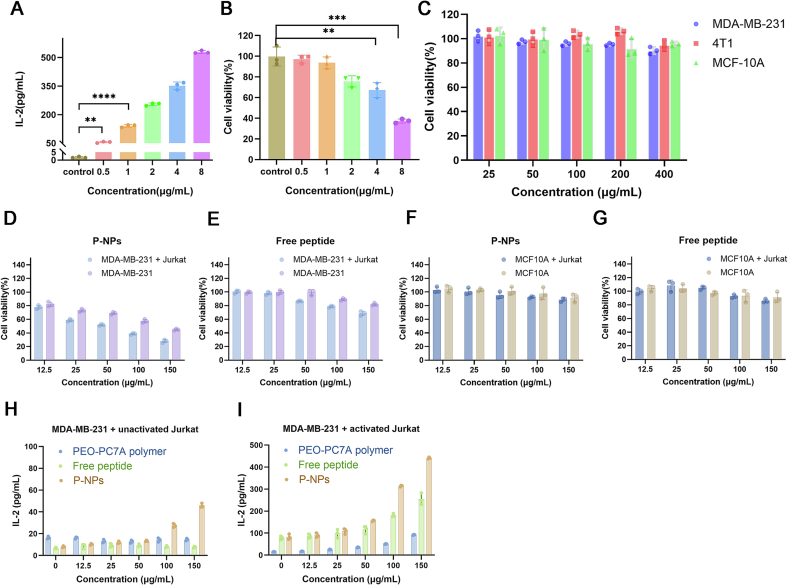
PHA-induced activation of Jurkat cells and P-NP-mediated anti-tumor effects in co-culture assays. (A) IL-2 secretion levels in Jurkat cell supernatants after 48 h treatment with varying concentrations of PHA. IL-2 production increases with PHA dose, indicating enhanced T cell activation. (B) Viability of Jurkat cells after 48 h of PHA treatment, assessed by CCK-8 assay. (C) Viability of MDA-MB-231, 4 T1, and MCF10A cells after 48 h exposure to various concentrations of blank PEO-PC7A nanoparticles, showing minimal cytotoxicity of the carrier. (D-E) Viability of MDA-MB-231 tumor cells after 48 h co-culture with Jurkat T cells and treatment with different concentrations of peptide-loaded P-NPs (D) and free peptide (E) at corresponding concentrations. (F-G) Viability of MCF10A tumor cells after 48 h co-culture with Jurkat T cells and treatment with different concentrations of peptide-loaded P-NPs (F) and free peptide (G) at corresponding concentrations. (H—I) IL-2 levels in co-culture supernatants after 48 h treatment with P-NPs or free peptide in MDA-MB-231 co-cultured with unstimulated Jurkat or PHA-stimulated Jurkat cells, measured *via* ELISA. Statistical significance: **P < 0.01; ***P < 0.001; ****P < 0.0001, compared to untreated control.

The results indicated a direct correlation between PHA concentration and IL-2 secretion, with even the lowest dose (0.5 μg/mL) eliciting a response greater than the untreated control, thus confirming successful activation (Fig. 4A). While cell viability was unaffected at lower concentrations, a marked decrease was observed at PHA concentrations of 2 μg/mL and higher, which we attribute to overstimulation or induced cellular apoptosis (Fig. 4B).

Based on these results, a PHA concentration of 1 μg/mL was selected as optimal for activating Jurkat cells in our co-culture assays. This concentration achieved a strong activation signal, without significant cytotoxicity. This balance ensured that T cells were both robust and functionally competent for subsequent interaction with tumor cells.

#### 3.3.2 *In vitro* cytotoxicity assays

To ensure the safety of the delivery platform, we first assessed the cytocompatibility of the blank PEO-PC7A nanoparticles in three cell lines, including MDA-MB-231, 4 T1, and MCF-10 A. Across all cell models, over 90 % viability was maintained after 48 h exposure to up to 400 μg/mL, confirming excellent biocompatibility of the polymeric carrier (Fig. 4C).

Next, we evaluated the antitumor effects of the free anti-PD-L1 peptide and peptide-loaded P-NPs. To differentiate between direct and immune-mediated effects, experiments were performed with and without pre-activated Jurkat cells. As shown in Fig. 4D-G, without Jurkat cells, neither free peptide nor P-NPs exerted significant toxicity in MDA-MB-231 or MCF-10 A cells. However, in co-culture with activated Jurkat cells, P-NPs significantly enhanced tumor cell killing compared to free peptide, confirming that the nanoparticle formulation amplified immune-mediated cytotoxicity (Fig. 4D).

When applied to MCF-10 A/Jurkat co-cultures, both free peptide and P-NPs showed minimal cytotoxic effects (Fig. 4F and G), consistent with low PD-L1 expression in MCF-10 A(Bajor et al., 2022; Hsu et al., 2018). The results indicated the specificity of P-NPs toward tumor cells through PD-1/PD-L1 blockade, rather than nonspecific toxicity.

#### 3.3.3 P-NPs potentiate T Cell Activation *via* a dual Mechanism

To further investigate the mechanism underlying the enhanced cytotoxicity observed in co-culture experiments, we quantified IL-2 secretion from both PHA-activated and unstimulated Jurkat T cells. As shown in Fig. 4H, unstimulated Jurkat cells didn't secret high level of IL-2 after treatment with PEO-PC7A polymer, free peptide, or P-NPs, consistent with the lack of basal immune activation in the absence of external stimuli.

In contrast, upon PHA stimulation, Jurkat cells showed restored immune responsiveness. In detail, treatment with blank PEO-PC7A nanoparticles did not significantly increase IL-2 levels relative to PHA alone (Fig. 4I), indicating that the carrier alone did not act as a non-specific mitogen. Treatment with free peptide led to a modest but reproducible increase in IL-2, consistent with its role as a PD-L1 antagonist that relieved checkpoint-mediated suppression. Notably, the combination of PHA stimulation and P-NPs induced the highest IL-2 secretion across all tested concentrations, markedly exceeding the levels observed with either component alone.

The superior efficacy of the P-NPs can be attributed to a dual mechanism of action. First, the anti-PD-L1 peptide blocked the PD-1/PD-L1 immune checkpoint pathway, thereby alleviating T cell exhaustion and restoring effector function. Second, the PEO-PC7A polymeric carrier itself contributed to innate immune activation by stimulating the cGAS-STING pathway. Given that STING is not functionally expressed in Jurkat cells, the innate immune stimulation likely originated from co-cultured tumor cells, leading to the secretion of type I interferons and pro-inflammatory cytokines that further promote T cell activation. The elevated IL-2 levels served as a downstream readout of this combined adaptive and innate immune engagement. This dual-action strategy not only enhanced tumor cytotoxicity but may also contributed to systemic immune activation and durable antitumor immunity. While future studies employing STING-deficient tumor cells or alternative carriers are warranted, the current results validated the rational design of our P-NPs and their potential as a platform for synergistic cancer immunotherapy.

### 3.4 *In vivo* animal studies

#### 3.4.1 Biodistribution analysis of peptide-loaded P-NPs

**Fig. 5:**
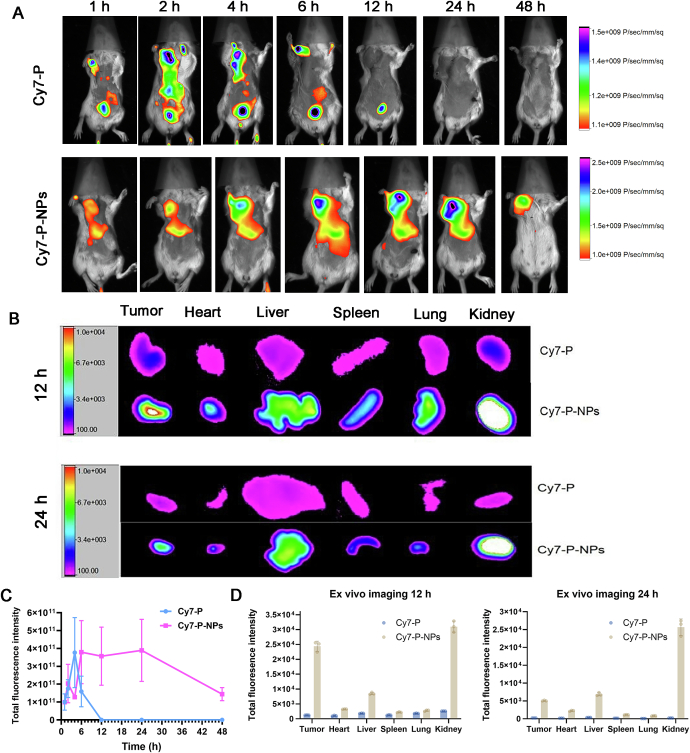
*In vivo* biodistribution of Cy7-labeled peptide delivered *via* P-NPs compared to free peptide in 4 T1 tumor-bearing mice. (A) Representative *in vivo* fluorescence images of tumor accumulation at different time points. (B) *Ex vivo* images of tumors and major organs at 12 h and 24 h post-injection. (C) Quantitative analysis of *in vivo* fluorescence intensities in tumors tissues over time (*n* = 3). (D) Quantitative analysis of *ex vivo* fluorescence intensities in major organs and tumor tissues at 12 h and 24 h post-injection (n = 3). Data are presented as mean ± SD.

To elucidate the tumor-targeting capability of our P-NPs and their potential to enhance therapeutic accumulation, we investigated their biodistribution *in vivo* compared to the free peptide. For real-time fluorescence tracking, the peptide was labeled with a near-infrared dye (Cy7), yielding Cy7-labeled peptide (Cy7-P) and Cy7-labeled peptide-loaded nanoparticles (Cy7-P-NPs). These were intravenously injected into 4 T1 tumor-bearing mice. Whole-body *in vivo* live imaging was conducted at 1, 2, 4, 6, 12, 24, and 48 h post-injection (Fig. 5A), and *ex vivo* imaging of tumors and major organs were performed at 12 h and 24 h (Fig. 5B).

As shown in Fig. 5A, free peptide rapidly accumulated in the tumor within 1 h and peaked at 4 h, followed by a rapid decline in fluorescence. In contrast, Cy7-P-NPs exhibited delayed but sustained tumor accumulation, with fluorescence peaking between 6 and 12 h and maintaining high levels up to 24 h. Quantitative analysis of tumor fluorescence (Fig. 5C) confirmed these trends, demonstrating that P-NPs prolonged circulation and tumor retention compared to free peptide.

*Ex vivo* fluorescence imaging at 12 h and 24 h (Fig. 5B) further validated these observations. At both time points, P-NPs showed significantly higher fluorescence in tumor tissue compared to free peptide. While some uptake in liver and spleen was observed for both formulations, which was likely due to reticuloendothelial system (RES) mediated clearance. Notably, the tumor-to-organ ratio was higher for P-NPs, particularly at 24 h. By 48 h, fluorescence in both tumor and major organs declined, indicating systemic clearance of the nanoparticles, potentially through hepatic and renal pathways.

Overall, these biodistribution studies demonstrated that P-NPs significantly improved the pharmacokinetic profile of the therapeutic peptide. The nanoparticle formulation enabled sustained tumor enrichment, prolonged circulation, and enhanced retention, all of which were critical for maximizing therapeutic efficacy while minimizing off-target exposure. The result also indicated that the 12–24 h window post-injection represented the optimal timing for therapeutic intervention.

#### 3.4.2 *In vivo* antitumor efficacy

**Fig. 6:**
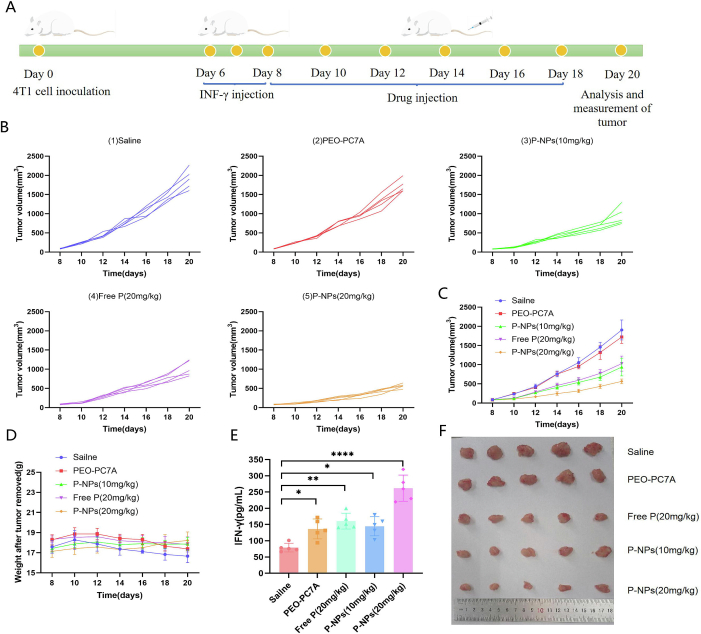
Therapeutic efficacy of P-NPs in a 4 T1 murine tumor-bearing mice model. (A) Schematic of tumor inoculation, drug administration, tumor volume monitoring, and analysis timeline. (B, C) Individual (B) and average (C) tumor growth curves under different treatments: saline, blank NPs (no peptide), free peptide (20 mg/kg), low-dose P-NPs (10 mg/kg peptide), and high-dose P-NPs (20 mg/kg peptide). (D) Representative images of excised tumors from each group at study end. (E) Body weight change curves of mice during treatment. (F) IFN-γ levels in tumor tissue at the end of treatment for each group, measured by ELISA. *P < 0.05; **P < 0.01; ****P < 0.0001, *vs.* saline control.

Encouraged by our *in vitro* and biodistribution results, we assessed the therapeutic efficacy of P-NPs in a 4 T1 tumor-bearing mouse model (Fig. 6A). The tumor growth profiles revealed that both the free peptide and P-NPs significantly inhibited tumor progression compared to the saline control group (Fig. 6B and C). However, the P-NP formulation achieved a superior therapeutic outcome. Mice receiving high-dose P-NPs (20 mg/kg peptide) exhibited the most pronounced tumor inhibition. Furthermore, the low-dose P-NPs group (10 mg/kg peptide) also outperformed the free peptide at equivalent or higher dosages, suggesting the enhanced therapeutic potency provided by nanoparticle encapsulation. The size of the excised tumors at the endpoint visually confirmed these quantitative differences among groups, with the smallest tumors observed in the high-dose P-NP group (Fig. 6D).

Systemic tolerability was monitored throughout the treatment period. No significant body weight loss was observed in any treatment group, indicating that neither the free peptide nor the P-NPs induced overt toxicity at the administered doses (Fig. 6E). This favorable safety profile is critical for the potential clinical translation of the therapy.

To investigate the underlying immunological mechanisms, we measured the intratumoral concentration of IFN-γ, a pivotal cytokine in antitumor immunity. As shown in Fig. 6F, all active treatments increased IFN-γ levels compared to the saline control. However, the P-NP-treated groups induced a significantly more potent cytokine response. Tumors from the high-dose P-NP group exhibited the highest IFN-γ levels, confirming that the enhanced tumor control was associated with a more robust local immune activation.

#### 3.4.3 Immunohistochemical Analysis of Tumor Immune Microenvironment

The antitumor mechanism of P-NPs was further explored by means of immunohistochemistry. Tumor tissues were stained for markers indicative of cytotoxic T lymphocyte infiltration (CD8), T cell activation (Granzyme B, GZMB), regulatory T cells (Foxp3), and STING pathway activation (phosphorylated TBK1).

**Fig. 7:**
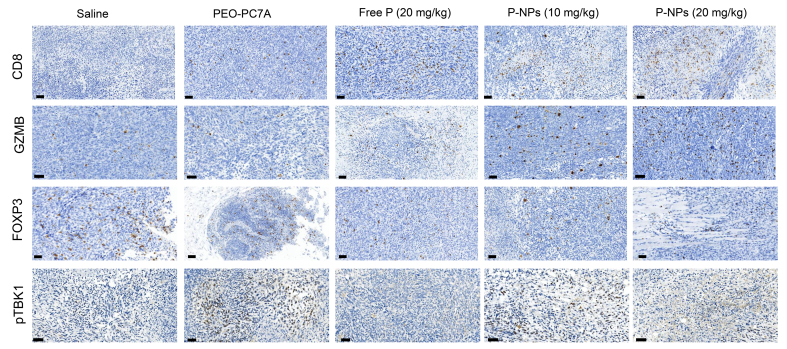
Evaluation of immunotherapeutic mechanisms *in vivo*. Immunohistochemical staining of tumor sections from different treatment groups. CD8 is for CD8^+^ T cell infiltration. Granzyme B (GZMB) expression is a marker of CD8^+^ T cell activation. Foxp3^+^ is for regulatory T cells. Phosphorylated TBK1 (pTBK1) expression is indicative of STING pathway activation. Scale bar = 50 μm.

As shown in Fig. 7A, tumors from the high-dose P-NP treated group showed a high density of CD8^+^ T cells infiltrating the tumor parenchyma, significantly more than in tumors from the free peptide group or untreated controls. Similarly, the GZMB level was the highest in the high-dose P-NP treated group, indicating the activation of CD8^+^ T cells (Fig. 7B). This correlated with the enhanced antitumor activity we observed and indicated that P-NP treatment led to better recruitment or expansion of cytotoxic T cells in the tumor. For Foxp3^+^ Tregs (Fig. 7C), we observed a decrease in tumor treated with high-dose P-NP. A lower Treg presence is consistent with a more favorable environment for anti-tumor immunity.

To assess innate immune activation *via* the cGAS-STING pathway, we investigated the levels of phosphorylated TBK1 (p-TBK1). If the cGAS-STING pathway is activated, increased phosphorylation of TBK1 can be observed. Notably, blank PEO-PC7A nanoparticles alone induced detectable p-TBK1 expression (Fig. 7D), confirming STING pathway engagement. This is in accordance with a modest but clear increase in IFN-γ. The highest levels of p-TBK1 were observed in the high-dose P-NP group, indicating enhanced innate immune activation through synergistic action of the carrier and peptide. This dual-mode strategy, combining peptide-mediated T cell restoration with polymer-driven innate immune stimulation, appears to effectively convert the tumor into a more immune-responsive microenvironment, thereby amplifying the overall therapeutic effect.

Our results demonstrated a structure-to-function validation, from PC7A protonation and pH-dependent disassembly, to accelerated peptide release, to enhanced T-cell activation and *in vivo* efficacy. Unlike conventional pH-responsive carriers that only modulate release, the PC7A domain actively participated in immunotherapy by engaging STING, thereby converting the carrier from a passive vehicle into an immuno-active matrix. This dual action streamlined formulation with no separate adjuvant, focused immune stimulation to acidic compartments, and provided a mechanistic basis for the observed synergy between checkpoint blockade and innate immune priming. These features differentiate our platform from prior pH-responsive systems and suggest a generalizable route to multifunctional, stimulus-coupled immuno-nanomedicines.

In summary, the synergistic immunotherapy approach validated the rationale of our nanoparticle design, providing strong preclinical evidence for further translational development.

## 4 Conclusion

In this study, we successfully developed a multifunctional nanocarrier platform that integrated a previously developed anti-PD-L1 peptide with a pH-sensitive amphiphilic block copolymer, PEO-PC7A. Our approach exploited the unique pH-triggered disassembly of PEO-PC7A nanoparticles to achieve controlled and targeted peptide release specifically within the acidic tumor microenvironment. This targeted release not only enhanced the peptide's stability, extended its systemic circulation time, improved tumor accumulation, and reduced off-target effects. Beyond its role as a delivery vehicle, the PEO-PC7A copolymer also served as an intrinsic activator of the STING signaling pathway, facilitating the conversion of an immunosuppressive tumor microenvironment into an immunostimulatory one. This dual-action system not only restored T cell-mediated antitumor immunity through PD-1/PD-L1 blockade but also reprogrammed the immunosuppressive tumor microenvironment by stimulating innate immune response. Together, these combined mechanisms produced a robust antitumor effect in both *in vitro* and *in vivo* models, providing strong preclinical evidence for the therapeutic potential of this platform.

## CRediT authorship contribution statement

**Mengjie Rui:** Writing – original draft, Supervision, Methodology, Formal analysis, Conceptualization. **Haidan Tang:** Writing – original draft, Visualization, Methodology, Investigation, Formal analysis. **Lianglai Gao:** Writing – original draft, Visualization, Methodology, Investigation, Formal analysis. **Yujiao Hu:** Writing – original draft, Investigation. **Wenyan Liang:** Writing – original draft, Investigation. **Yinfeng Li:** Writing – original draft, Investigation. **Chunlai Feng:** Writing – review & editing, Supervision, Project administration, Funding acquisition, Conceptualization.

## Declaration of competing interest

The authors declare that they have no known competing financial interests or personal relationships that could have appeared to influence the work reported in this paper.

## Data Availability

Data will be made available on request.
